# Influence of different data cleaning solutions of point‐occurrence records on downstream macroecological diversity models

**DOI:** 10.1002/ece3.9168

**Published:** 2022-08-04

**Authors:** Petra Führding‐Potschkat, Holger Kreft, Stefanie M. Ickert‐Bond

**Affiliations:** ^1^ Biodiversity, Macroecology and Conservation Biogeography, Faculty of Forest Sciences University of Göttingen Göttingen Germany; ^2^ Department of Biology and Wildlife & UA Museum of the North University of Alaska Fairbanks Fairbanks Alaska USA

**Keywords:** automated data cleaning pipelines, data quality, expert data, GBIF, species distribution modeling

## Abstract

Digital point‐occurrence records from the Global Biodiversity Information Facility (GBIF) and other data providers enable a wide range of research in macroecology and biogeography. However, data errors may hamper immediate use. Manual data cleaning is time‐consuming and often unfeasible, given that the databases may contain thousands or millions of records. Automated data cleaning pipelines are therefore of high importance. Taking North American *Ephedra* as a model, we examined how different data cleaning pipelines (using, e.g., the GBIF web application, and four different *R* packages) affect downstream species distribution models (SDMs). We also assessed how data differed from expert data. From 13,889 North American *Ephedra* observations in GBIF, the pipelines removed 31.7% to 62.7% false positives, invalid coordinates, and duplicates, leading to datasets between 9484 (GBIF application) and 5196 records (manual‐guided filtering). The expert data consisted of 704 records, comparable to data from field studies. Although differences in the absolute numbers of records were relatively large, species richness models based on stacked SDMs (S‐SDM) from pipeline and expert data were strongly correlated (mean Pearson's *r* across the pipelines: .9986, vs. the expert data: .9173). Our results suggest that all *R* package‐based pipelines reliably identified invalid coordinates. In contrast, the GBIF‐filtered data still contained both spatial and taxonomic errors. Major drawbacks emerge from the fact that no pipeline fully discovered misidentified specimens without the assistance of taxonomic expert knowledge. We conclude that application‐filtered GBIF data will still need additional review to achieve higher spatial data quality. Achieving high‐quality taxonomic data will require extra effort, probably by thoroughly analyzing the data for misidentified taxa, supported by experts.

## Graphical abstract

This study examined how data cleaning tools differed in their resulting cleaned GBIF data and how this affected downstream macroecological diversity models. Taxonomic and spatial errors identified in the GBIF Ephedra data, filter categories: false positives.

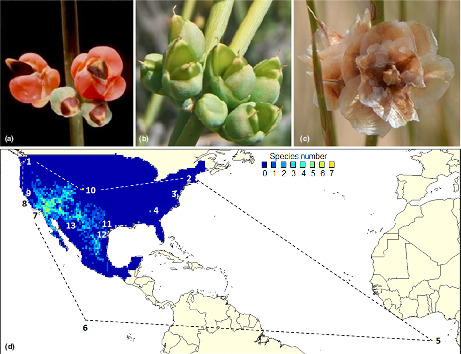

## 1 INTRODUCTION

Digitally accessible species records from global data‐sharing networks like the Global Biodiversity Information Facility (GBIF) provide the basis to address a wide range of biodiversity‐related questions in ecology, biogeography, and other disciplines (e.g., Guralnick et al., 2007; Meyer et al., 2016; Soberón & Peterson, 2004). Such databases and data‐sharing networks represent a valuable source of knowledge in which individual researchers and institutions worldwide invested considerable amount of time and resources (Baskauf et al., 2016; Guralnick et al., 2018; Wieczorek et al., 2012). However, since the circumstances and standards under which these records were collected and digitized are usually unknown, a user must assess whether the data quality provided meets the requirements of the research question (Beck et al., 2013; Sterner & Franz, 2017). Consequently, this demands data cleaning tools (hereafter: DC tool) to standardize data and identify and remove data errors. Thus, developing appropriate DC tools is a long‐standing goal of biodiversity informatics (e.g., Araújo & Guisan, 2006; Chapman et al., 2000; Kadmon et al., 2004).

Data errors occur mainly along three dimensions: taxonomy, space, and time (Meyer et al., 2016). They may significantly affect common downstream analyses such as the accuracy of species distribution models (SDMs, e.g., Gueta & Carmel, 2016, Tessarolo et al., 2017, Hijmans & Elith, 2019, Zizka et al., 2019). In the taxonomic dimension, resolving misspellings (Zermoglio et al., 2016) and reconciling the synonymy of taxonomic names (Alroy, 2002; Wortley & Scotland, 2004) pose a significant challenge. The related widespread and particularly challenging problem is misidentified specimens, estimated at 50% for tropical plant specimens (Goodwin et al., 2015) and ranging from 5% to nearly 60% in the Zoological Record database (Meier & Dikow, 2004). In the spatial dimension, errors in and low precision of coordinates, for example, from rounding of the decimal digits, swapped latitude and longitude, missing coordinates, or coordinates with zero‐values are common data quality problems (e.g., Otegui et al., 2013; Töpel et al., 2017; Yesson et al., 2007). Lower geospatial accuracy is frequently assumed for older records than for those collected more recently (Tessarolo et al., 2017; Zizka et al., 2020). Stropp et al. (2016) showed, for instance, that conspicuous records of flowering plants collected in Africa before the 1960s were filtered out due to poor data quality. Another issue associated with older records is that the probability increases that populations no longer exist at a given sampling location over time due to natural or anthropogenic reasons (Meyer et al., 2016).

Even for experts, identifying and resolving data quality issues manually is in many cases unfeasible, given that datasets typically contain thousands to millions of records. Therefore, selective DC strategies based on well‐explained instructions and automated DC tools that reproducibly generate high‐quality data are especially in high demand for inexperienced users (Zizka et al., 2019). Downstream applications such as conventional SDMs depend on these data quality (e.g., Araújo et al., 2019; Guisan et al., 2017; Raes & Aguirre‐Gutiérrez, 2018). Data scientists and biodiversity informaticians approached the development of DC solutions from several angles: (1) DC tools that generally solve thematically limited requirements, like retrieving, evaluating, formatting, completing, and organizing data. This type of DC solution was implemented in the widely used *Tidyverse* “umbrella” package (Wickham et al., 2019). The solution was also included in specialized packages such as *CoordinateClearer* (Zizka et al., 2019), *rgbif* (Chamberlain, 2020), and the GBIF web application (GBIF.org, 2020). (2) Manuals supporting the preparation of data for SDMs. Particular *R* packages are an integral part of such manuals (e.g., Chapman, 2005; Guisan et al., 2017; Hijmans & Elith, 2019). The manuals consist of verbal explanations and coded instructions, which the user can apply (e.g., per package *dismo*, Hijmans & Elith, 2020). While the newly developed and recently updated methods for automated cleaning of records are promising, their effect on commonly applied SDMs remains poorly examined (see Hijmans et al., 2017; Schmidt‐Lebuhn et al., 2013; Zizka et al., 2020).

Pipelines play an important role in the scientific domain when, for example, biodiversity data from different sources such as herbarium vouchers and observations need to be combined for analysis. In this study, we investigated the performance of six pipelines (P1 to P6) using various DC tools and how these pipelines affected downstream SDMs. We used North American *Ephedra* species as the model organisms (Ephedraceae, Gnetales; Cutler, 1939; Stevenson, 1993, Figure 2, A to C; Table S1) and GBIF as the data source. With over 2.1 billion species records worldwide, GBIF is the largest and one of the most frequented public providers of biodiversity data. It is often the primary data source for many researchers (Guralnick et al., 2018; Hobern et al., 2019; Zizka et al., 2020). Thus, we selected the GBIF records as input to the pipelines. In this context, we address three questions:

1. How do the pipelines differ in their performance? We expect that different DC tools will generate different result datasets.
2. How do differences in pipeline data affect downstream diversity models and maps (observed, predicted)? We expect the pipeline datasets to differ in the resulting models (single species and stacked SDMs, hereafter: S‐SDM) and maps.
3. How does the pipeline data—after being cleaned by the pipelines—differ from the expert data (observed and predicted), assuming that the expert data represent the most accurate *Ephedra* environmental and geographical range? We expect the quality of the pipeline data to differ from the expert data. The differences will be measurable (occurrences and correlations) in the models and maps.

We analyzed to which extent the data from the different pipelines led to different species constellations and numbers in the grid cells and visualized the differences in diversity maps created from S‐SDMs. Finally, we discuss how realistic the results from GBIF data and expert data reflect the environmental or geographical extent of the *Ephedra* species' ranges.

## 2 MATERIALS AND METHODS

In North America, *Ephedra* species are characteristic components of arid and semi‐arid regions of the southwestern USA and Mexico (Hollander & VanderWall, 2009; Loera et al., 2015). They occur from the Death Valley to about 2500 m in the Rocky Mountains (Stevenson, 1993). The species share a morphologically reduced, uniform growth habit with mostly leafless, photosynthetic stems (Ickert‐Bond & Renner, 2016). Specimens are collected frequently, as shown by the record numbers of the public providers (e.g., GBIF: 46,384 records worldwide), and high‐quality expert data are available for the New World species (Ickert‐Bond, 2003). The coordinates served as the proxy for the *Ephedra* species' characteristic locations (response variables), from which we developed species SDMs and genus S‐SDMs for North America.

We monitored changes in similarities and correlations using the validated records from P1 to P6 and the expert data (observed occurrences, hereafter: L1; Table 2). From L1, we developed L2 and L3 data of the North American *Ephedra* species and their occupied grid cells (per pipeline and the expert data). L2 included the grid cell numbers an *Ephedra* species occupied, and L3 counted the concurrent *Ephedra* species per grid cell. L4 data comprised the correlations of the observed occupied grid cells. The L5 data (pipeline and expert) included the predicted distribution in S‐SDMs across the pipelines and expert data (L2/L4, and L5: Spatial autocorrelation by Moran's *I* and correlation between two random variables by Pearson's *r*) (Figure 3).

### 2.1 Data pipelines

**FIGURE 1:**
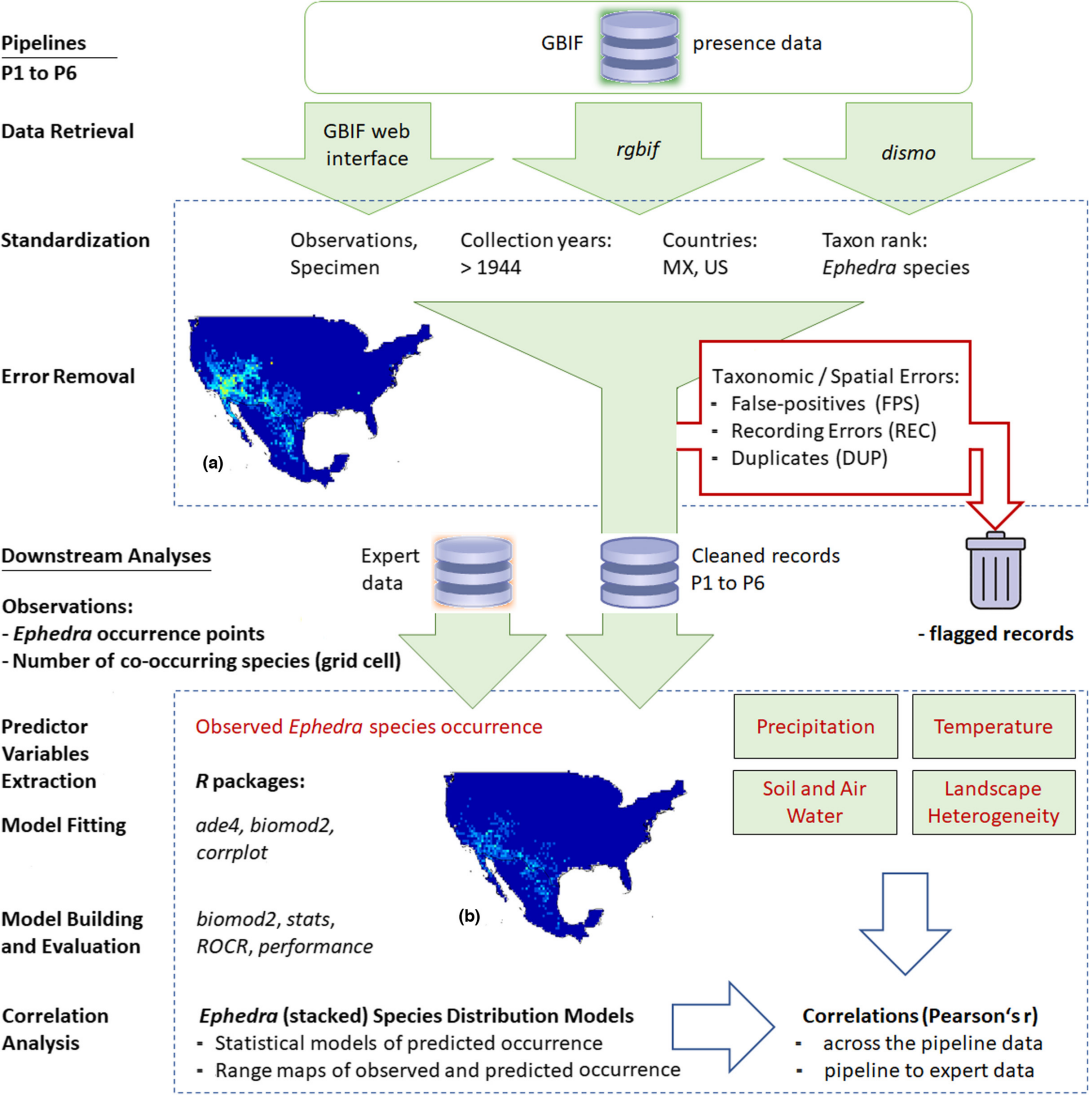
Workflow of the pipelines and the downstream analyses. The pipelines' part comprised the following sections: Data Retrieval, Standardization, and Error Removal. The Downstream Analysis featured the Predictor Variables Extraction, the Model Fitting, the Model Building (SDMs, S‐SDMs) and Evaluation, and the Correlation Analysis developed from the pipeline data P1 to P6 and the expert data. *R* packages used in the course of the workflow are in italics. (a) Observed species distribution from GBIF P1 data. (b) Observed species distribution from expert data. Filter categories: DUP, Duplicate records; FPS, False positives; REC, Recording Errors.

Ensuring comparability across six pipelines, the process chain of filters provided identical conditions to optimize the provider data (See Table 1, the filters of the pipelines). The chain consisted of (1) selecting and retrieving data from GBIF, (2) standardizing the records by filtering, and (3) correcting or removing data errors (Figure 1, Table 2). At each pipeline step, we employed one or more DC tools (e.g., Chapman, 2005; Hijmans & Elith, 2019; Zizka, 2019). The selected tools (e.g., GBIF web application, written instructions, or *R* packages) or their most recent updates were released between 2005 and 2020 and are free of charge. In some pipelines, the three steps were performed by one (“three‐in‐one”) DC tool. In the setup of the process chain, we followed the data cleaning recommendations given by the respective DC tool's authors and pertinent best‐practice guidelines (Araújo et al., 2019; Guisan et al., 2017).

**TABLE 1:** Pipeline filter summary for standardization and error removal

Categories	Filter	Requirement	Rationale
STD	Country range	Spatial	North America: Mexico and the USA
STD	Infraspecific rank	Taxonomic	Required rank: species (Claridge et al., 1997; Reydon & Kunz, 2019), infraspecific ranks (e.g., subspecies, hybrids) to be omitted.
STD	Collection years	Temporal	1945 to 2020, as older records are more likely to contain erroneous coordinates (Zizka et al., 2020).
STD	Basis of record	Consistency	Specimens and observations.
STD	Occurrence status	Consistency	Presence data.
FPS	Non‐North America‐native *Ephedra* species	Taxon	All non‐native *Ephedra* species that are allocated to the North American countries either by mistake or are artificially introduced, for example, to botanical gardens.
FPS/REC	Zero or missing coordinates	Spatial	Zeroes and missing values may represent records with data entry errors. Missing values will cause error messages in *ade4*.
REC	Longitude and latitude are equal	Spatial	Equal longitude and latitude may represent records with data entry errors.
DUP	Duplicate records	Consistency	Duplicate records that may represent, for example, record copy errors.
FPS	Country capitals	Spatial	Records that may contain the coordinates of the country capital.
FPS	Country centroids	Spatial	Records that may contain the centroid coordinates of the country.
FPS	GBIF headquarters	Spatial	Records that may contain the coordinates of the GBIF headquarters.
FPS	Biodiversity institutions	Spatial	Records that may contain the coordinates of biodiversity institutions where the herbarium voucher is stored.
FPS	Geographic outliers	Spatial	Geographic outliers that may represent misidentified specimens.
REC	Urban areas	Spatial	Records from urban areas that may represent old data or vague locality descriptions.
REC	dd.mm to dd.dd conversion errors	Spatial	Records with ddmm to dd.dd conversion error (misinterpretation of the degree sign as decimal delimiter).
REC	Rasterized collections	Spatial	Records with a significant proportion of coordinates that might have a low precision.
FPS	“Manual” removal of false positives	Consistency	False positives that have been overlooked by automated error removal, based on the knowledge that they are in the records.

*Note*: Categories: DUP, duplicate records; FPS, false positives; REC, recording errors; STD, standardization.

**TABLE 2:** Results of the pipelines' data cleaning performance, compared to the P0 benchmark dataset (summary table)

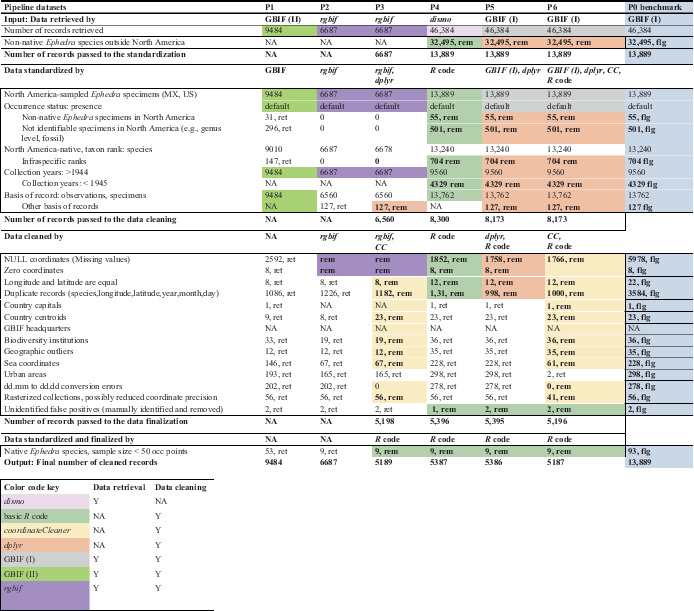

*Note*: The color‐coded cells of P1 to P6 datasets indicate the activity of a particular DC tool (color code see below). The blue cells of the P0 benchmark indicate the number of *Ephedra* records in GBIF, quantified by standardization and error category. Records which did not comply with the standardization conditions or were erroneous in the context of this study were flagged (flg). Since several standardization conditions and errors coincided in the same record, the number of removed records did not correspond to the sum of the identified errors. The P1, P2, and P3 data retrieval tools partially standardized the data and eliminated several errors (“three‐in‐one” tools). Thus, the number of records retrieved differed significantly from P4 to P6, and P0. The removed records in these pipelines could only be reconstructed as differences of subcategories (e.g., in‐scope countries, collection year, null and zero coordinates) in comparison to P0. The difference between P3 and P2 resulted from the added *dplyr* and *CC* packages, which increased standardization and removed still more erroneous records. Using the added packages ensured more insight into data cleaning.

Abbreviation: *CC* (→ P3/P6) = *R* package *CoordinateCleaner*.

We retrieved data from GBIF (gbif.org, 2020) on November 18, 2020, in four different ways: (1) The filter “*Ephedra* L.” (hereafter: GBIF (I)) retrieved 46,384 records for P5, P6, and the P0 benchmark data using the “three‐in‐one” GBIF web application (GBIF, 2020a). (2) The filter set “*Ephedra* L. specimens of North America, from 1945 to 2019” (hereafter: GBIF (II)) selected 9484 records for the P1 process chain using the web application (GBIF, 2020b). In both cases, the data were downloaded with the web application. (3) *rgbif*, a “three‐in‐one” tool, employed its integrated functionality to standardize the P2 and P3 data and retrieved 6687 GBIF records into the userspace. (4) *dismo* selected 46,384 GBIF records for P4 and retrieved them into the userspace. (Details see Table 2).

**FIGURE 2:**
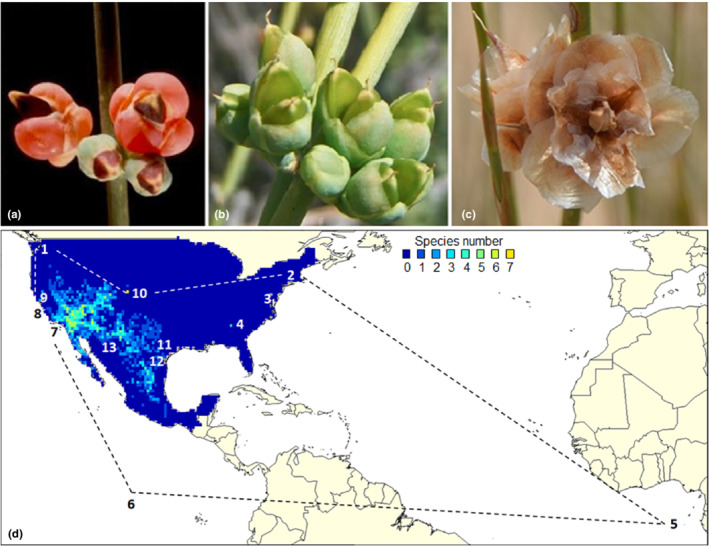
(a–c) North America‐native *Ephedra* specimens (female specimens with seeds). *Ephedra antisyphilitica, E. nevadensis*, and *E. trifurca* (left to right). (d) Examples of taxonomic and spatial errors identified in the *Ephedra* data. Filter categories of the following markers: False positives. Markers 1, 8, and 9 were specimens from shops in Seattle and Berkeley. Markers 3, 4, 10, and 11 were non‐native species from botanical gardens and scientific institutes. Marker 2 pointed to a North America‐native species at the University of Connecticut, NY. Markers 5 to 7 showed coordinate errors that the verbatim locality description can only identify. The species at markers 12 and 13 were misidentified, as the documented species do not occur naturally at these localities. The data for the map derived from the P1, post‐cleaning (L3, number of co‐occurring species). Color coding of the map: P1 observed distribution (see Figure 4).

We created the P0 data for comparison. It served as the benchmark of standardization and errors, delivered by the GBIF data, which the DC tools could have removed in the pipelines. However, P0 was not itself a pipeline nor was it part of any pipeline. We performed an inventory of the dataset and the data errors that might influence the quality of the downstream models (Table 2, P0 column). Using P0, we could identify questionable records and the degree of feasibility to which each pipeline removed such records. After data retrieval, further data cleaning was performed in P3, P4, P5, and P6 by basic *R* code (R Core Team, 2013), the *dplyr* package (of *Tidyverse*, Wickham et al., 2019), and the *CoordinateCleaner* (Zizka, 2019; Zizka et al., 2019), in different combinations (Table 2). We selected records of taxon rank “species” (Claridge et al., 1997; Reydon & Kunz, 2019), filtered for North America (Mexico, USA), and collection years 1945 to 2020 (Zizka et al., 2020). As the basis of records, we selected specimens and observations. During error removal, we focused on taxonomic and spatial errors (Meyer et al., 2016), such as non‐native specimens, missing or zero values, and sea coordinates. We also removed false‐positive records reporting, for example, occurrences at biodiversity institutions, and geographic outliers. From the P0 evaluation, we were aware of two false‐positive occurrences (Figure 2, Marker 2) hidden in the data. We found these errors challenging to be recognized by any tool. Therefore, we removed one of these errors in P4, and two in P5 and P6, using basic *R* code. As coordinates with three or fewer decimal places often indicate they were obtained from grid maps (Zizka et al., 2019), we permitted only validated coordinates with no less than four decimal places. However, this precision was not required for the modeling. The *CoordinateCleaner* identified specimens of urban areas and flagged them for scrutiny. We searched for duplicates based on the variables: species, coordinates, and collection date, respectively, and removed them. Finalizing the process chains, we excluded native species for which the sample size was lower than 50 occurrences to avoid biased models and maps (Guisan et al., 2017; Hijmans & Elith, 2019). (Usage of the tools in the pipelines, see Table 2). At the end of the pipelines, we examined the retained records and errors in the pipelines' datasets in comparison to P0 (data at L1).

### 2.2 Downstream analysis

Data from examination of physical herbarium specimens and field studies (Ickert‐Bond, 2003) represented the most realistic environmental and geographical range (“gold standard”, Araújo et al., 2019) of the genus *Ephedra* in North America. The expert dataset comprised 4081 records of New World Ephedra specimens from herbaria with large holdings of *Ephedra* in both North and South America (e.g., ARIZ, ASU, HUH, NY, RM, SGO, SI, TEX, UC, UNAM, US; herbarium acronyms according to Thiers, 2022). A total of 704 records of 12 *Ephedra* species (L1) were selected for North America; however, they were not processed in a pipeline. We applied standardization conditions only for comparability. The records contained confirmed taxa, examined coordinates, and detailed locality descriptions comparable to field‐collected data. We considered an overlap of 90 records of 13,889 from GBIF and the expert dataset negligible. As *Ephedra* is adapted to dry environments, we imported 19 temperature and precipitation variables from the CHELSA climatology (Karger et al., 2017), elevation data as a proxy for landscape heterogeneity (GMTED, 2020), and plant‐available water data (Zhang et al., 2018). From their habitat description (e.g., Cutler, 1939; Stevenson, 1993), we assumed the selected environmental data being ecologically relevant.

For the SDMs and S‐SDMs, we created a grid of 4017 cells across Mexico and the USA (30 arc minutes, WGS84) using wrld_simple (*R* package *maptools*, Bivand et al., 2022) and *raster* (Hijmans et al., 2017). The grid size reasonably showed the co‐occurring species, which was not the case on different scales. We aggregated the environmental data to the grid resolution (*sp* package, version 1.4‐5, Bivand et al., 2013; Pebesma & Bivand, 2005) and extracted the values for each occurrence (*raster*; Hijmans & van Etten, 2021). We built a presence‐absence table, creating a random selection of pseudo‐absences for each *Ephedra* species using the *R* package *biomod2* (Thuiller et al., 2016). We tested the localities where *Ephedra* species were not recorded (*R* package *ecospat*, Di Cola et al., 2017). We anticipated environmental conditions to cause absence (Loera et al., 2015; Stevenson, 1993), making sure that the localities used for fitting the model represented the requirements of the species across North America (Training area, Guisan et al., 2017). We summed up the species present in the grid cells as the number of co‐occurring species. (L2, L3).

We identified the contributing predictors (using *R* packages *ade4*, Bougeard & Dray, 2018 and *corrplot*, Wei et al., 2017). From the 21 variables, we selected a subset of reasonably uncorrelated variables per species using *biomod2* (Table S2; Guisan et al., 2017; Thuiller et al., 2016). Reasonably uncorrelated refers to being below the recommended threshold of 0.7 (Dormann et al., 2013). As goodness‐of‐fit evidence, we used the Akaike Information Criterion (AIC; Johnson & Omland, 2004), and Tjur's *R* ^2^ (Coefficient of Discrimination for binary outcomes; *R* package *performance*, Lüdecke et al., 2021) to identify the variables with the highest impact (Table S2). Finally, we fitted logistic regression models for the *Ephedra* occurrences using glm as the model and “binomial” as the distribution family. The threshold value of a high‐performance index (0.9, Guisan et al., 2017) was used to evaluate the predictive accuracy of the model, particularly the Receiver Operating Characteristic Curve (ROC) and the area under the curve (AUC) (*R* packages *biomod2,* Thuiller & Lafourcade, 2019, and *ROCR*, Sing et al., 2015). We stacked the predictions of the 12 *Ephedra* species resulting from the different pipelines as well as the expert data to S‐SDMs (without using thresholds; Biber et al., 2020; Calabrese et al., 2014; Guisan et al., 2017). The correlations between the observed and the predicted *Ephedra* occurrences informed how strongly the differences between the pipelines and the expert data affected the respective SDMs and S‐SDMs (L5).

We inspected spatial autocorrelation (L2/L4: grid occupation, L5: predicted distributions) using the Moran's *I* coefficient (*R* package *spdep*, Bivand et al., 2015). We computed the correlations of the observed and predicted *Ephedra* occurrences in two pipelines (the least cleaned data, P1, and the most cleaned data, P6) and the expert data using Pearson's *r* (*R* package *rstatix*, Kassambara, 2020). Ultimately, we visualized them as map pairs (Figure 4); and to adequately represent the species richness in the maps, we chose 11 breaks (*R* package *classInt*, Bivand, 2022) for the maximum possible co‐occurring species.

## 3 RESULTS

The GBIF web interface using GBIF (I) filters and *dismo* retrieved 46,384 unstandardized and uncleaned, globally distributed *Ephedra* datasets. The GBIF web interface using GBIF (II) filters retrieved 9484 partially standardized *Ephedra* records from North America. *rgbif* retrieved 6687 somewhat standardized specimen records from North America and already removed significant spatial errors. (Download results see Table 2). The three tools stopped after the data retrieval.

### 3.1 P0 benchmark data

About 13,889 P0 records represented the unstandardized and uncleaned GBIF North American *Ephedra* data. A total of 1979 specimens were collected or observed in Mexico (14.2%) and 11,910 in the USA (85.8%). The majority of species records consisted of North America‐native *E. viridis* (19.0%), *E. aspera* (14.4%), *E. californica* (14.1%), *E. nevadensis* (13.3%), *E. trifurca* (11.9%), and *E. torreyana* (8.7%), a total of 81.4% for six species. Another six native species, *E. antisyphilitica* (4.4%), *E. funerea* (2.4%), *E. fasciculata* (1.8%), *E. pedunculata* (1.5%), *E. compacta* (1.3%), and *E. cutleri* (1.1%) totaled 12.5%. The remaining 6.1% were non‐native (55 taxonomic false positives of South American and Eurasian origin) or indeterminate specimens (499 specimens of genus *Ephedra* L.). Several standardization conditions and errors coincided in the same record. Thus, the number of removed records did not correspond to the sum of the identified errors. About 5187 records (37.3%) were flagged as fit‐for‐use for the downstream analyses. Around 8702 records (63.7%) were marked for removal due to one or more significant errors. Missing coordinates (5978 records, 43.1%) represented the majority of identified data errors, followed by the sampling year (4329 records, 31.1%, were older than 1945) and the duplicate records (3584 records, 25.8%). About 220 records showed coordinates in bodies of water. With two exceptions, the non‐native *Ephedra* species were, for example, found in botanical gardens and scientific institutes (e.g., Atlanta Botanical Garden; Figure 2d, locality markers 3, 4, 10, and 11). As a few non‐native species contain medicinally active substances, they were reported with two records from a shop in Berkeley (*E. sinica*, Figure 2d, locality markers 8 and 9) and one record from an herbal product shop in Seattle (*E. sinica*, Figure 2d, locality marker 1). We detected *E. nevadensis* at the University of Connecticut (Figure 2d, locality marker 2), yet this species is native to the Southwestern United States. Three records revealed misplaced taxa by comparing the verbatim locality description with the coordinates. These errors were not identified by a tool, only by scrutiny. Locality marker 12 referenced a misidentified specimen (*E. distachya*, Figure 2d) that does not naturally occur in Coahuila, Mexico. The specimen that locality marker 13 referenced (*E. trifurcata*, Figure 2d) might be a misspelling of *E. trifurca* (P0 results, see Table 2, Table S1).

### 3.2 Expert data

Five hundred seventy‐seven of 2251 specimens were collected or reviewed from Mexico (25.3%) and 1674 specimens from the US (75.4%). After standardization, 704 records remained (210 records of Mexican specimens, 494 records of US specimens). After standardization, the majority of records (65.2%) were allocated to *E. aspera* (22.3%), *E. trifurca* (21%), *E. fasciculata* (11.4%), and *E. antisyphilitica* (10.5%). The other eight species, *E. viridis* (7.1%), *E. californica* (5.4%), *E. torreyana* (5.1%), *E. funerea* (4.5%), *E. compacta* (3.7%), *E. pedunculata* (3.3%), *E. nevadensis* (2.3%), and *E. cutleri* (1.4%) totaled 32.8% of the standardized records. The remaining 14 records (2%) were of other taxonomic ranks.

### 3.3 Effects of differences in the pipeline data on diversity models

P1 and P2 were partly standardized in their process chain. GBIF (II) of P1 met four out of five standardization requirements. Explicit error removal did not occur; however, P1 implicitly removed 3386 missing coordinate records as a side effect of the standardization. It left 2592 missing coordinates records, 296 indeterminate records, and 33 South American and Eurasian species in the P1 dataset. P2's *rgbif* met three standardization requirements, but the resulting data still contained infraspecific ranks. *rgbif* standardized the P2 data partly, using the parametrized standardization criteria, and, in addition, the built‐in error exclusion parameter of invalid coordinates was employed. Except for excluding missing values in the coordinates, P2 removed no other spatial errors.

P3, P4, P5, and P6 continued their respective process chains. The pipelines removed between 43.1% and 45.3% of all spatial error types (e.g., the complete subset of 5986 missing coordinates records, see Table 2). P3 used the *dplyr* and *CoodinateCleaner*, providing 5189 records to the downstream analyses. In P4, we fully standardized the data, using instructions explained in a tutorial (Hijmans & Elith, 2019) and basic *R* code. P4 provided 5387 records to the downstream analyses. In P5, we standardized the data and removed errors, using basic *R* code and the *dplyr*. P5 provided 5386 records to the downstream analyses. P6 used instructions from Chapman (2005) translated to basic *R* code and *dplyr* functionality to handle taxonomic errors. The *CoordinateCleaner* removed spatial errors. P6 identified 5187 fit‐for‐use records for the downstream analyses. Due to not meeting the sampling size criteria, we manually removed *Ephedra coryi* records from the pipelines. At the end of the pipelines, the records for the downstream analyses varied considerably and ranged from 9484 (P1) to 5187 (P6) (L1) (Table 2).

**FIGURE 3:**
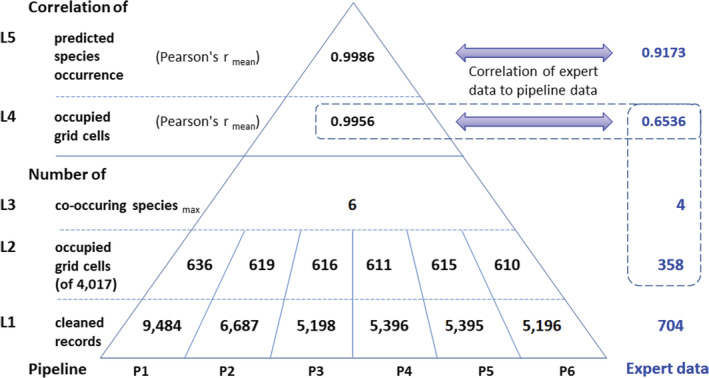
Information condensing pyramid of the pipelines and the expert data (L1 to L5: Condensing levels of the data). The data show an increasingly higher correlation from the bottom to the top of the pyramid, which results from data transformations into an increasingly higher condensed species occurrence information state. The 704 expert data occurrences (L1) were allocated into 358 grid cells (L2, with a maximum of four co‐occurring species, L3). The correlation of 0.6536 (L4, mean Pearson's *r* of pairings [P1 to P6/expert]) was compared to the mean of the pairings P1 to P6. At this level (L4), the minimum Pearson's *r*‐value of the occupied grid cells from pipeline data was .9920 (pair: P1/P6), and the maximum Pearson's *r* value was .9999 (pair: P4/P5). At the L5 level, the minimum Pearson's *r* value was .9951 (pair: P1/P6), and the maximum Pearson's *r* value was 1.0000 (pair: P4/P5). Dashed box: Expert data comparison numbers, L2 to L4.

**FIGURE 4:**
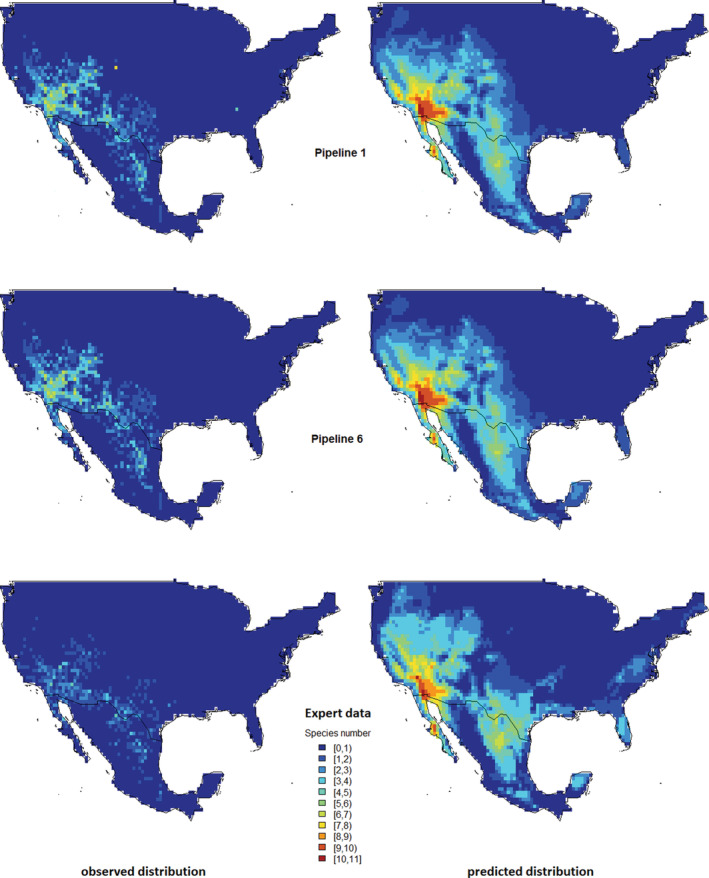
Stacked species distribution maps based on cleaned GBIF data from pipelines P1, P6, and expert data. Depicted are the maps of the least cleaning P1 and the most cleaning P6 that show only minor differences (the maps from the other pipeline data are close to P6). The control data map from the expert data shows differences to the pipelines. Left: Observed distribution (L2 data). Point‐occurrences after passing the pipelines, allocated to grid cells of a stacked range map of all *Ephedra* species. The expert map shows less occupied grid cells (*n* = 358) than P1 (*n* = 636) resulting in a smaller range. Right: Map of the predicted probability of species from S‐SDMs (L5 data). The color keys show highly correlated patterns of each data quality (P1, P6, and expert data: 0 to 12 species, Pearson's *r* = .9173).

The cleaned datasets differed by 4288 (P1 vs. P6), and the number of occupied grid cells by 26 grid cells (maximum). We observed similarly clustered occupancy patterns in the distribution maps regardless of the pipeline since most records were allocated to the same grid cells per species. The occupied grid cells in the stacked *Ephedra* range maps varied between 636 and 610 (P1 vs. P6 data). Comparisons of highly correlated occupied grid cells (mean Pearson's *r* across the pipelines: .9956) were confirmed by highly correlated maps of observed *Ephedra* distribution with well‐defined clusters (Figure 3, and Figure 4, P1 and P6 map pairs). Moran's *I* confirmed the spatially clustered patterns of the *Ephedra* species (observed P1/P6 Moran's *I*: 0.144, observed expert data's Moran's *I*: 0.087, *p*‐value: significant) (L2/L4). *Ephedra californica* occurrences occupied identical grid cells across all six pipelines; therefore, the Pearson correlation coefficient was 1. For the other 11 *Ephedra* species, the occupancy of the grid cells varied slightly across the pipelines, depending on the respective pipelines compared. For example, in *E. fasciculata*, P1 differed from P6 with 49 versus 53 occupied grid cells (92.5% identical occupancy), while the occupancy in P2 and P3 in *E. antisyphilitica* was again identical (Pearson's *r* = 1). The evaluation of the S‐SDMs showed that the grid cell occupancy patterns (observed occurrences) continued in the species distribution maps (predicted occurrences). Correlograms based on residual analysis are listed in Figure S2.

Post‐pipelines, we found that the *ade4* indicated coordinates with missing values as invalid in records containing this error type, hence, may also be regarded as a testing point for missing values in the coordinates. (Note that we did not intervene in the data cleaning in P1 by GBIF (II). Thus, records with missing values in coordinates were preserved).

The final number of predictors for the species ranged from 4 (*Ephedra aspera*) to 10 (*Ephedra viridis*) (Table S2). The area under the curve (AUC) scored from 0.9355 (*Ephedra antisyphilitica*) to 0.9990 (*Ephedra nevadensis*) (AUC mean: 0.9825). The AIC decreased to a stable minimum value in the variable's combination tests, indicating the best possible model performance compared to the other variable combinations. Therefore, we considered our models as adequately accurate to describe the distribution of the *Ephedra* species with the identified explanatory variables. The differences in the pipelines had a minor effect on the correlations, models, and maps at L4 and L5. At level L4, the mean Pearson's *r* of the occupied grid cells across the pipelines was 0.9956 (P1/P6 pair: 0.9920, minimum; P4/P5 pair: 0.9999, maximum). The high correlation led to maps of observed *Ephedra* distribution that showed also only insignificant differences (Figure 4, P1 and P6 observed distribution). Across the six pipelines, the predicted probability of occurrence from the S‐SDMs indicated high correlations (mean Pearson's *r* = .9986, Figure 3, L5). Figure 4 displays the maps of the predicted distribution based on the S‐SDMs.

### 3.4 Differences between pipeline data and expert data

The 704 expert data occurrences (L1) were allocated into 358 grid cells, with a maximum of four co‐occurring species (L3). Across the pipelines, 294.5 of the average 630.5 grid cells (46.7%) showed occupancy by one species, compared to 265 of 358 grid cells (74.0%) of the expert data. 42.6 of the grid cells showed occupancy by four species (6.7%), compared to the maximum of four species (1.1%) of the expert data. Ten grid cells showed occupancy by the maximum of six species (1.6%) in the pipeline data (L2).

The correlations differed clearly between the pipelines and the expert data. At level L4, the mean Pearson's *r* of the occupied grid cells for pipeline data correlated to the expert data was .6536 (L4: Figure 3). The correlation of the predicted occurrence probabilities in the S‐SDMs showed a mean Pearson's *r* of .9173. Across the different pipelines and the expert data, the observed diversity in the maps from the S‐SDMs showed a large *Ephedra* diversity center in Southern California that continued to the North into Arizona and Nevada, and to the South into the states of Baja California and Sonora, Mexico with a predicted *Ephedra* diversity greater than seven species. A second diversity center emerged across the state of Texas, USA, and continued into the states of Chihuahua, Coahuila, Nuevo Léon, and Tamaulipas, Mexico, with a predicted *Ephedra* diversity of up to seven species. (L5). The diversity patterns in the expert data, although similar in shape, were less distinct (Figure 4).

## 4 DISCUSSION

We analyzed the data cleaning performance of six different pipelines for digital point‐occurrence records and their effects on species distribution models, a common downstream application in macroecology. The six pipelines differed significantly in the number of accepted species, errors removed, and remaining records for analysis (Table 2, Table S1). For example, P6 removed the most significant number of records, approximately twice as many records as the least cleaning pipeline P1. Data from P1 differed from the other group by hosting 17 non‐native species in addition to the 12 natives, all of which were removed by the other pipelines. P1 also retained false‐positive coordinates (e.g., sea, country capitals and centroids, biodiversity institutions, herbal shops), geographic outliers, and duplicates, which were removed to different degrees by the pipelines of the other group (Table 2) (Question 1).

Due to the low complexity of the data cleaning environment, P1 and P2 required only little effort to get their pipelines installed. Both pipelines did not achieve the standardization and error elimination anticipated to reduce unwanted effects in the downstream analyses. P1 identified potential shortcomings in the data only in a few cases due to the limited options of the GBIF filter application. In contrast, P3 to P6 were more demanding in the required know‐how, mainly when using the *R* packages and preparing the respective user environments but offered a more substantial functionality (Table 2). The *R* packages performed the data cleaning well for coordinate errors that rendered records unusable for use in diversity models. Generalist packages like the *dplyr* and specialists like the *CoordinateCleaner*, especially in combination, reliably identified problematic records with missing values and false‐positive occurrences such as biodiversity institutes or country centroids. Accurate distribution data are essential for any SDM and the many comparable downstream analyses (Araújo & Guisan, 2006; Chapman et al., 2000; Kadmon et al., 2004; Zizka et al., 2020). Therefore, the main aim of well‐designed pipelines is to efficiently and automatedly generate cleaned data tailored to the specific research question (Zizka et al., 2020; Table 1). We mainly focused on comparing the outcomes of different pipelines that used well‐known data retrieval or DC tools to answer this question. The standardization filters served to unify the record structure across the pipelines. Although older herbarium vouchers or observations are as valuable as recent vouchers since they may document both a historical status and biodiversity changes over time (Meyer et al., 2016), the “collection year, older than 1945” filter, for example, was implemented to standardize the data but also to reduce expected general coordinate imprecisions up‐front. However, removing taxonomic and spatial errors was at the core of the pipeline data for the model fitting and model building and the respective tools.

### 4.1 Influence of different data cleaning solutions on downstream analyses

Removing the non‐native species, which consisted of only a few specimens, reduced the number of cleaned records only slightly (per species and overall). The non‐native *Ephedra* species had no noticeable effect in the occupied grid cells as co‐occurring species. They were concentrated in a few places and in small numbers of species only (P1, Figures 3 and 4: observed distribution). The low level of differences was confirmed by reasonably high correlation coefficients, which continued to even higher correlation coefficients regarding the predicted probability of species in S‐SDMs (L1 to L5: Figure 3). Removing the missing value records in the pipelines was essential for the downstream analyses. The model fitting tool issued error messages when identifying any in the provided data (*ade4*). Although we included the duplicate records filter in determining the number of duplicate records in the data, duplicate records did not affect the fitted models (Question 2).

The tested pipelines offer automated data cleaning in a standardized and reproducible manner. Pipeline P1 supports all users but produces data that still contain serious taxonomic and spatial errors. In contrast, the pipelines P2 to P6, which help users with some programming experience (Zizka et al., 2019, 2020), produce data qualities where many errors were eliminated and which seem suitable for diversity model use (SDMs and S‐SDMs).

### 4.2 Significant differences of the expert data and the GBIF data

The P1 data differed noticeably from the expert data, for example, in the species composition (P1 data: 29 species vs. expert data, and P2 to P6 data: 12 species), the number of records per species, the number of occupied grid cells after the observations were allocated to gridded range maps (Figure 3, L2), and the number of co‐occurring species. P2 to P6 differed less from the expert data. (Question 3). The aim of collating data for SDMs is to avoid bias and inaccuracies in taxonomic and distribution data, and an effective means of overcoming bias and inaccuracies is to build data from field studies (Araújo et al., 2019; Chapman, 2005). Well‐maintained expert data support both the aims and provide an alternative to field studies. A less maintained data alternative, biodiversity records from GBIF, are free of charge but with limitations in data quality due to several known and unknown errors. Expert and GBIF data form the data layer (Bakshi, 2012; Vetter, 1990). However, the critical difference between expert data and GBIF data is that the expert data may be used unprocessed as input to the data modeling workflow as there are no data errors to be expected. For the GBIF data, an additional data cleaning process chain needs to be included in the workflow so that the data modeling can be meaningfully linked to the data layer. Consequently, a user of GBIF data always has to plan for an additional effort for the data cleaning design, which includes the functional structure of the target data that is fit for use, and a pipeline to obtain it (Wirth & Hipp, 2000; Zizka et al., 2019).

### 4.3 A major issue: misidentified specimens that still hide in the dataset

Comparing the quantities of the GBIF pipelines' analysis data and the expert data shows that the expert data are roughly 11.8% or about one‐eighth of the GBIF data (mean). From this ratio, we may assume that there are still many errors in the pipeline data, hence, the visible differences in the maps (Figure 4). This point opens the question of how realistic the GBIF data is. No pipeline detected taxonomic issues such as misidentifications or false positives like non‐native specimens in the data due to a lack of information about their distributional status. For differently determined specimens of the same origin, given to other institutes and handled in isolation from their parent specimens, Nicolson (2019) provided a technical solution. We used expert know‐how to assess the likeliness of taxonomic identities in recorded localities as there presently is no tool that possesses this functionality (Figure S1). Developing a tool that resolves this issue might be challenging considering the many names, from synonyms to misspellings (Zermoglio et al., 2016). A correction method that was already introduced is that a data owner directly changes false positives identified in individual cases by notifying the provider. Generally, with the present interfaces to GBIF, it cannot be avoided that misidentified taxa enter into the databases by, for example, citizen scientists. Interfaces that prevent taxonomic or spatial errors before entering a public provider must be designed.

## 5 CONCLUSION

Our results suggest that the P1 data show more differences from P2 to P6 data than within this group. Depending on the pipeline, one‐third (P1) to two‐thirds (P6) of the GBIF records were classified as unsuitable for biodiversity analyses. Importantly, differences in the pipeline data did not translate into significant differences in downstream SDMs and S‐SDMs, suggesting remarkable robustness of these analyses toward data cleaning differences. The increasingly condensed information from the occurrence data led to ever stronger correlations across the pipelines. Three aspects emerged from the study. First, data from the GBIF web application require further cleaning. Second, the *R* packages reliably removed incorrect or dubious coordinates. Therefore, choosing the right DC tools depends on the researcher's skills. Third, it is challenging to identify misidentified specimens in the public data providers. To overcome this difficulty, we suggest new processes to identify misidentified specimens or prevent new misidentified specimens from being entered into the public data providers. Consequently, programmers developing new data cleaning packages should consider the requirements for data cleaning, notably as the *CoordinateCleaner* eliminates most spatial errors.

## AUTHOR CONTRIBUTIONS

**Petra Führding‐Potschkat:** Conceptualization (lead); data curation (lead); formal analysis (lead); funding acquisition (lead); investigation (lead); methodology (lead); project administration (lead); resources (equal); software (equal); supervision (lead); validation (equal); visualization (lead); writing – original draft (lead). **Holger Kreft:** Conceptualization (supporting); data curation (supporting); formal analysis (supporting); funding acquisition (supporting); investigation (supporting); methodology (supporting); project administration (supporting); resources (equal); software (equal); supervision (supporting); validation (equal); visualization (supporting); writing – original draft (supporting). **Stefanie M. Ickert‐Bond:** Conceptualization (supporting); data curation (supporting); formal analysis (supporting); funding acquisition (supporting); investigation (supporting); methodology (supporting); project administration (supporting); resources (equal); supervision (supporting); validation (equal); visualization (supporting); writing – original draft (supporting).

## CONFLICT OF INTEREST

The authors involved in the preparation of this manuscript have no conflicts of interest to declare.

## Supporting information


Appendix S1

## Data Availability

P0 benchmark data and pipelines P4 (*dismo*‐retrieved), P5, and P6 data: 46,384 worldwide distributed *Ephedra* records from GBIF, https://doi.org/10.15468/dl.2eg5ab. (GBIF, 2020a). Pipeline P1 data, filtered by the GBIF web application: 9484 North America‐distributed *Ephedra* records from GBIF, https://doi.org/10.15468/dl.r2cg62. (GBIF, 2020b). Pipelines P2 and P3 data (*rgbif*‐retrieved): 6687 North America‐distributed *Ephedra* records from GBIF, https://datadryad.org/stash/share/QspgKk8RRlEXK6grxNgdzde8KmofVOJC4_N6cfIY_bQ. North American *Ephedra* Expert data, 704 North America‐distributed *Ephedra* records, https://datadryad.org/stash/share/7X7EDIZLIgVLjkyF0XJoqYEvOER9k3q8vDic2CZN2jE.
