# Supplementary material for: Influence of different data cleaning solutions of point‐occurrence records on downstream macroecological diversity models
Source: Ecol Evol. 2022 Aug 4;12(8):e9168. doi: 10.1002/ece3.9168 (PMC9351331; doi:10.1002/ece3.9168)
Supplement: Supplementary file 1 — Appendix S1 [file ECE3-12-e9168-s001.docx]

Table S1. Summary of the *Ephedra* species at the end of the pipelines (L1, record numbers per species). We retained the North America native species (Stevenson 1993) in the box for the downstream analyses and removed species outside the box, depending on exclusion criteria (Table 1, e.g., from other Geographies (GEO): EAs = Eurasia, SAm = South America). P0 data included all *Ephedra* records allocated to North America and served as the uncleaned control data to which we compared the other pipelines P1 to P6.

| ***Ephedra* species sums** |  | **9,484** | **6,687** | **5,198** | **5,396** | **5,395** | **5,196** | **13,889** |
| --- | --- | --- | --- | --- | --- | --- | --- | --- |
| **Taxon** | **GEO** | **P1** | **P2** | **P3** | **P4** | **P5** | **P6** | **P0** |
| *Ephedra antisyphilitica* Berland. ex C.A. Mey. | NAm | 375 | 211 | 185 | 187 | 187 | 184 | 612 |
| *Ephedra aspera* S. Watson | NAm | 1,478 | 1,166 | 835 | 920 | 919 | 837 | 1,994 |
| *Ephedra californica* S. Watson | NAm | 1,325 | 1,045 | 846 | 854 | 854 | 846 | 1,959 |
| *Ephedra compacta* Rose | NAm | 152 | 128 | 117 | 119 | 119 | 117 | 183 |
| *Ephedra cutleri* Peebles | NAm | 116 | 96 | 64 | 64 | 64 | 64 | 158 |
| *Ephedra fasciculata* A. Nelson | NAm | 184 | 158 | 119 | 129 | 129 | 119 | 245 |
| *Ephedra funerea* Coville & C.V.Morton | NAm | 198 | 146 | 113 | 113 | 113 | 113 | 328 |
| *Ephedra nevadensis* S. Watson | NAm | 1,264 | 952 | 666 | 672 | 672 | 664 | 1,845 |
| *Ephedra pedunculata* Engelm. ex S.Watson | NAm | 103 | 75 | 66 | 70 | 70 | 66 | 211 |
| *Ephedra torreyana* S. Watson | NAm | 811 | 571 | 435 | 445 | 445 | 435 | 1,210 |
| *Ephedra trifurca* S. Watson | NAm | 1,094 | 849 | 683 | 731 | 731 | 682 | 1,658 |
| *Ephedra viridis* Coville | NAm | 1,857 | 1,281 | 1,060 | 1,083 | 1,083 | 1,060 | 2,632 |
| *Ephedra coryi* E. L. Reed | NAm | 53 | 9 | 9 | 9 | 9 | 9 | 93 |
| *Ephedra miocenica* Wodehouse (fossil) | NAm |  |  |  |  |  |  | 2 |
| *Ephedra* L. (indeterminates) | NAm | 296 |  |  |  |  |  | 499 |
| *Ephedra* hybrid | NAm |  |  |  |  |  |  | 9 |
| *Ephedra* form and variety | NAm | 147 |  |  |  |  |  | 196 |
| *Ephedra altissima Desf.* | EAs | 2 |  |  |  |  |  | 4 |
| *Ephedra distachya* L. | EAs | 3 |  |  |  |  |  | 4 |
| *Ephedra equisetina* Stapf | EAs | 3 |  |  |  |  |  | 3 |
| *Ephedra fedtschenkoae* Paulsen | EAs | 1 |  |  |  |  |  | 1 |
| *Ephedra fragilis* Desf. | EAs | 1 |  |  |  |  |  | 3 |
| *Ephedra gerardiana* Wallich ex C. A. Mey. | EAs | 4 |  |  |  |  |  | 4 |
| *Ephedra major* Host | EAs | 1 |  |  |  |  |  | 1 |
| *Ephedra monosperma* J. G. Gmel. *ex* C. A. Mey. | EAs | 1 |  |  |  |  |  | 1 |
| *Ephedra przewalskii* Stapf | EAs | 2 |  |  |  |  |  | 2 |
| *Ephedra regeliana* Florin | EAs | 1 |  |  |  |  |  | 1 |
| *Ephedra sinica* Stapf | EAs | 3 |  |  |  |  |  | 8 |
| *Ephedra americana* Humb. & Bonpl*.* ex Willd*.* | SAm | 2 |  |  |  |  |  | 3 |
| *Ephedra andina* Poepp & Endl. | SAm |  |  |  |  |  |  | 2 |
| *Ephedra chilensis* C. Presl. | SAm | 2 |  |  |  |  |  | 2 |
| *Ephedra frustillata* Miers | SAm | 1 |  |  |  |  |  | 1 |
| *Ephedra triandra* Tul. | SAm |  |  |  |  |  |  | 2 |
| *Ephedra trifurcata* Zöllner | SAm | 3 |  |  |  |  |  | 11 |
| *Ephedra tweedieana* C. A. Mey | SAm | 1 |  |  |  |  |  | 2 |

Geographies, GEO: EAs, Eurasia; NAm, North America; SAm, South America.

Table S2. Uncorrelated CHELSA climatology variables (Karger et al. 2017) and plant-available water (PAWM), used to fit and build the *Ephedra* diversity models (twelve NAm Ephedra species; L4 and L5 data).

|  | ***E. antisyphilitica*** | ***E. aspera*** | ***E. californica*** | ***E. compacta*** | ***E. cutleri*** | ***E. fasciculata*** | ***E. funerea*** | ***E. nevadensis*** | ***E. pedunculata*** | ***E. torreyana*** | ***E. trifurca*** | ***E. viridis*** |  |
| --- | --- | --- | --- | --- | --- | --- | --- | --- | --- | --- | --- | --- | --- |
| **PAWM** |  |  |  |  |  |  |  |  |  |  |  |  | plant-available water |
| **bio2** |  |  |  |  |  |  |  |  |  |  |  |  | Mean diurnal range |
| **bio4** |  |  |  |  |  |  |  |  |  |  |  |  | Temperature seasonality |
| **bio5** |  |  |  |  |  |  |  |  |  |  |  |  | Max. temperature of the warmest month (°C) |
| **bio6** |  |  |  |  |  |  |  |  |  |  |  |  | Min Temperature of Coldest Month (°C) |
| **bio7** |  |  |  |  |  |  |  |  |  |  |  |  | Temperature annual range (°C) |
| **bio8** |  |  |  |  |  |  |  |  |  |  |  |  | Mean temperature of wettest quarter (°C) |
| **bio10** |  |  |  |  |  |  |  |  |  |  |  |  | Mean Temperature of Warmest Quarter (°C) |
| **bio11** |  |  |  |  |  |  |  |  |  |  |  |  | Mean Temperature of Coldest Quarter (°C) |
| **bio13** |  |  |  |  |  |  |  |  |  |  |  |  | Precipitation of wettest month |
| **bio14** |  |  |  |  |  |  |  |  |  |  |  |  | Precipitation of driest month |
| **bio15** |  |  |  |  |  |  |  |  |  |  |  |  | Precipitation seasonality |
| **bio16** |  |  |  |  |  |  |  |  |  |  |  |  | Precipitation of wettest quarter |
| **bio17** |  |  |  |  |  |  |  |  |  |  |  |  | Precipitation of driest quarter |
| **bio18** |  |  |  |  |  |  |  |  |  |  |  |  | Precipitation of Warmest Quarter |
| **bio19** |  |  |  |  |  |  |  |  |  |  |  |  | Precipitation of Coldest Quarter |


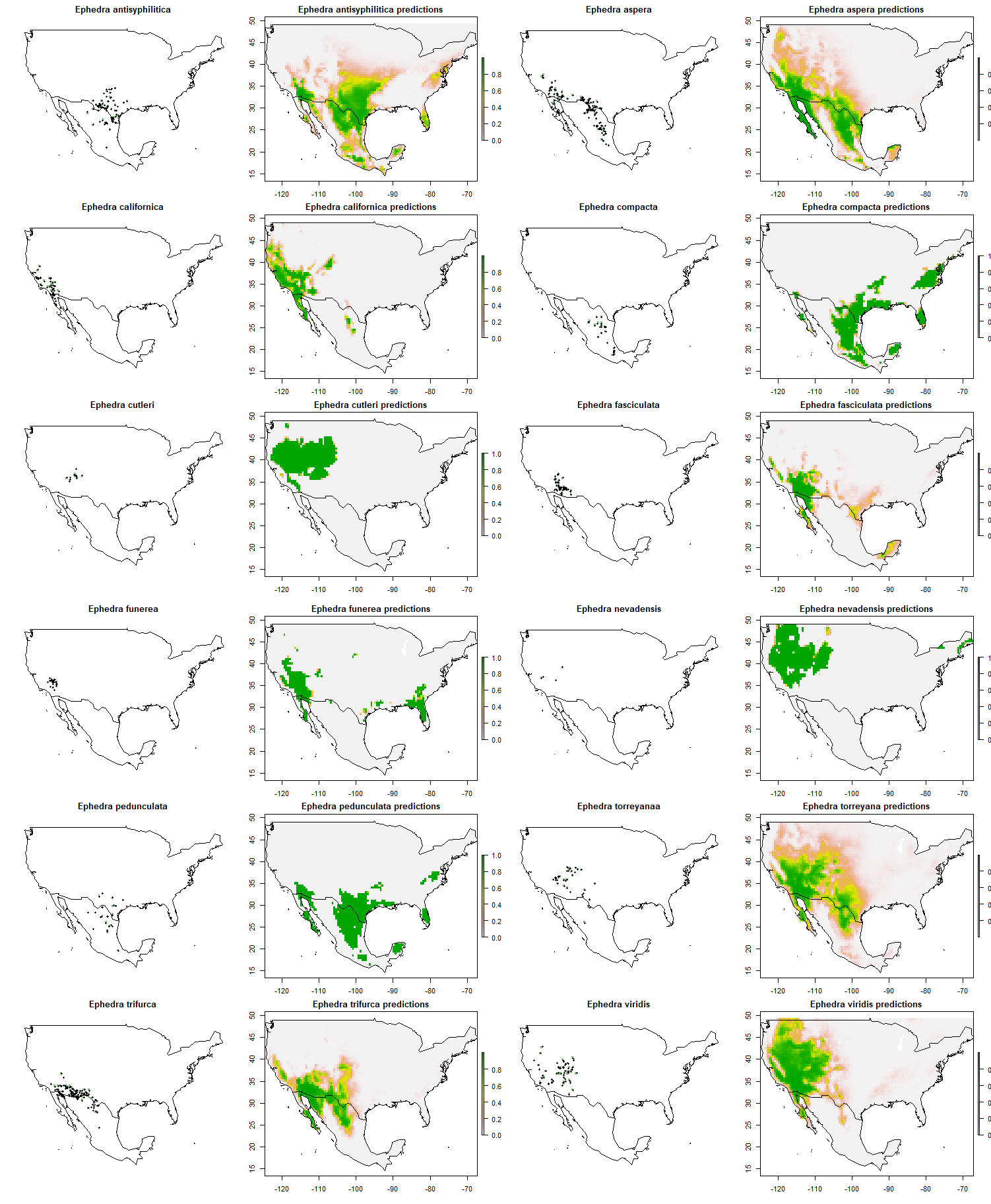


Figure S1. Observed occurrences and predicted ranges of each North American *Ephedra* species, from L5 expert data.


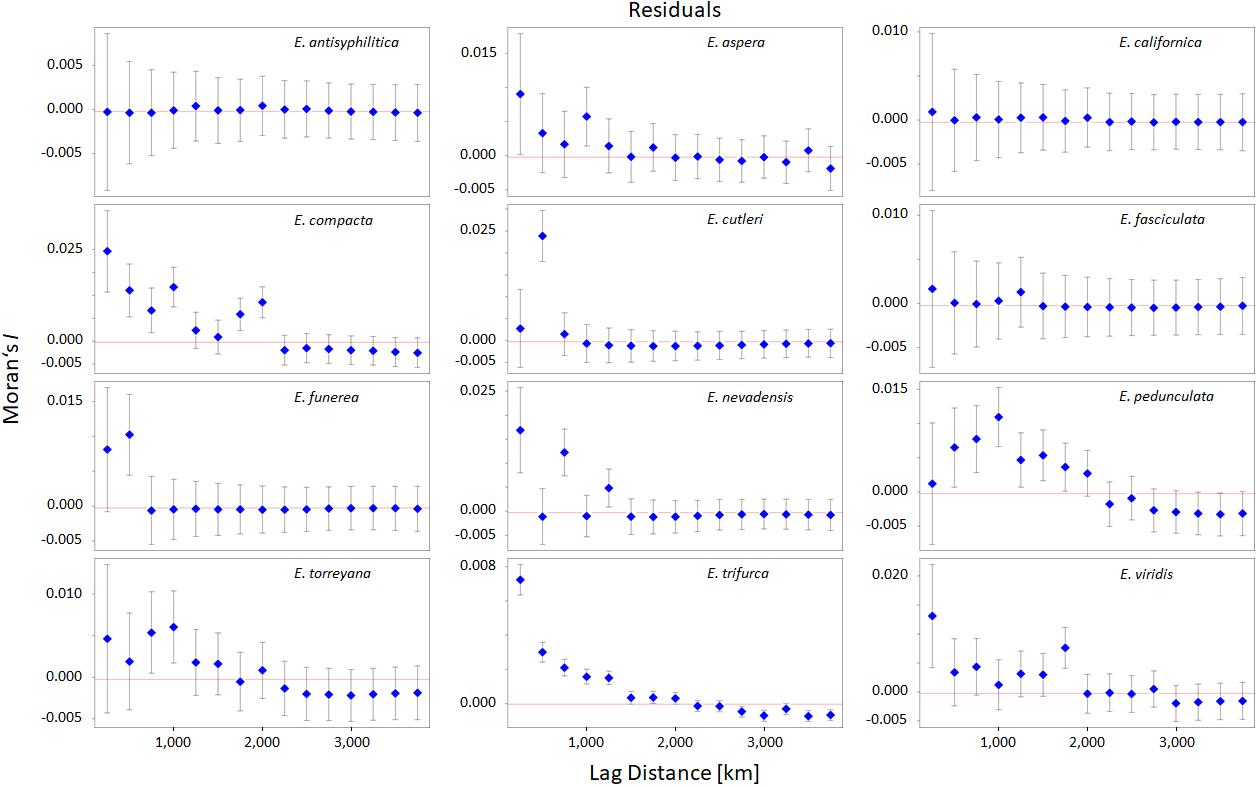


Figure S2. Spatial correlograms using Moran's *I* of raw species occurrence and residual variation after fitting the examined environmental variables at a grain size of 0.5, example from L5 expert data (*R* package *spdep*, Bivand et al. 2015). All correlograms are significant (p<0.0005), with the exception of *E. antisyphilitica* (p=0.20).
